# A Multi-Fault Diagnosis Method for Sensor Systems Based on Principle Component Analysis

**DOI:** 10.3390/s100100241

**Published:** 2009-12-29

**Authors:** Daqi Zhu, Jie Bai, Simon X. Yang

**Affiliations:** 1 Laboratory of Underwater Vehicles and Intelligent Systems, Shanghai Maritime University, Shanghai, 200135, China; 2 The Advanced Robotics and Intelligent Systems Laboratory, School of Engineering, University of Guelph, Guelph, ON. N1G 2W1, Canada; E-Mail: syang@uoguelph.ca

**Keywords:** multi-fault diagnosis, principal component analysis, signal reconstruction, fault detection, fault isolation

## Abstract

A model based on PCA (principal component analysis) and a neural network is proposed for the multi-fault diagnosis of sensor systems. Firstly, predicted values of sensors are computed by using historical data measured under fault-free conditions and a PCA model. Secondly, the squared prediction error (SPE) of the sensor system is calculated. A fault can then be detected when the SPE suddenly increases. If more than one sensor in the system is out of order, after combining different sensors and reconstructing the signals of combined sensors, the SPE is calculated to locate the faulty sensors. Finally, the feasibility and effectiveness of the proposed method is demonstrated by simulation and comparison studies, in which two sensors in the system are out of order at the same time.

## 1. Introduction

Since the principal component analysis (PCA) model can effectively reduce the dimension of input data, extract useful information and improve the efficiency of data analysis, it has been widely used in pattern recognition, signal processing, data compression, fault monitoring and many other fields [1–4]. The PCA model has been studied in depth in the field of fault diagnosis, especially in some complicated systems [4–7]. With the development of neural networks, some PCA diagnosis models based on artificial neural networks such as the Error Back Propagation (BP) neural network [8], the radial basis function (RBF) neural network [9,10] and the self organizing mapping (SOM) neural network [11] have been extensively studied in recent years. However, all the current fault diagnosis models aim at studying single-fault sensor systemd and rarely discuss multi-fault diagnosis (which is a common issue in the practical application). In addition, regarding the mentioned neural network PCA models, the structures are complicated, the parameters are difficult to design and the convergences are not so perfect. All of these hamper the practical use of the models. Compared to single-fault sensor diagnosis system, the real-time demand for multi-fault sensor diagnosis is higher and the calculations are more complicated, and consequently the PCA fault diagnosis method has rarely been applied to multi-fault sensor systems.

A rapid PCA fault diagnosis model for multi-fault sensor diagnosis systems based on a credit assigned cerebellar model articulation controller (CA-CMAC) is presented in this paper. As a result of the rapid convergence characteristics of the CA-CMAC, the real-time characteristics of the PCA fault diagnosis model are improved. The faulty sensors can be isolated by reconstructing the combined sensors’ signals, and thus a rapid diagnosis of multi-fault sensor systems is realized.

This paper is organized as follows. In Section 2, the conventional CMAC algorithm and CA-CMAC are introduced. The proposed PCA-based multi-fault sensor system diagnosis model is presented in Section 3. To illustrate the effectiveness of the proposed method, a simulation example is given in Section 4 and a comparison study is shown in Section 5. Finally, some concluding remarks are made in Section 6.

## 2. CMAC Algorithms

### 2.1. Conventional CMAC Algorithm

The CMAC neural network algorithm [12,13], which is built by imitating the behavior by which cerebella control human limbs, was proposed by Albus. It is a local approximating network with characteristics such as linear structure, simple algorithm, rapid learning speed and capability of dealing with uncertain knowledge. Also it has a generalization capacity. The basic idea of CMAC is storing the learning data (knowledge) in the overlapped memory cells (memory space). It contains two operations. The first is to output the network results, and the second is to adjust the weights (stored knowledge).

**Figure 1.**
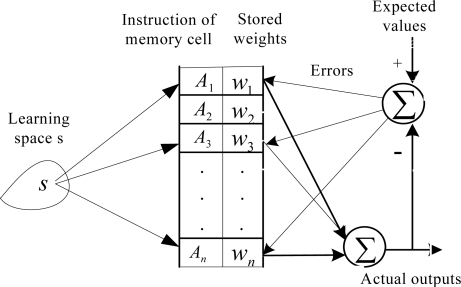
Basic structure diagram of a CMAC.

Its basic structure is shown as Figure 1, *S* is *n*-dimensional input space, *A* is the address of memory cells and *w* is the stored weights in memory cells. Each input *s_i_* activates the unit of memory space *A* to A^*^. The sum of connected weights which A^*^ corresponds to is the output:

(1) $$y_{i}=\sum_{j=1}^{N}\;w_{j}\;a_{j}\;(x),\;\;\;\;\;\;\;\;\;\;\;i=1,...,m$$

In Equation (1), *a_j_* is the memory cell activation flag. For the activated units, *a_j_* = 1, otherwise, *a_j_* = 0. For weight learning and adjusting of the network, supposed *S* is a state, *w_j_*(*t*) in Equation (1) is the weight stored in the *j*-th memory cell after *k*-th iteration. In conventional CMAC algorithm, errors are distributed to all the activated units averagely, so *w_j_*(*k*) can be updated as follows:

(2) $$w_{j}\;(t)=w_{j}\;(t-1)+\frac{\alpha }{m}\;a_{j}\;\left(\bar{y_{s}}-\sum_{j=1}^{N}\;a_{j}\;w_{j}\;(t-1)\right)$$

In Equation (2), $\bar{y_{s}}$ is the desired outputs of state *S*, $\sum_{j=1}^{N}\;a_{j}\;w_{j}\;(t-1)$ is the actual outputs of state *S*, *α* is a learning constant and *m* is the number of activated memory cells in some state. Only the weights of memory cells that have been activated will be updated.

### 2.2. CA-CMAC Algorithm

In the conventional CMAC algorithm, the errors are averagely distributed into all the activated memory cells. We know that the weights of CMAC has included the former learned knowledge after *k*-1 times of iteration, however not each addressed hypercube has the same learning experience, which leads to the differences in the reliability of each addressed memory cell weight and each addressed cell has a different contribution to errors. That’s to say, the same credit assignment should not exist in the *m* memory cells. If these differences are ignored and all the memory cells acquire the same amounts of errors, the errors produced by the state that has not been learned will cause “corrosion” to the former learned information, and in the network learning process, the desired data can only be gained after many learning cycles.

In order to improve the learning efficiency of CMAC and avoid the “corrosion” effect, the errors should be distributed in accordance with the memory cells’ credibility. However, no effective methods have been developed to decide which cell should take more responsibilities for the current errors. In other words, no good methods have been proposed to decide the credibility of the memory cells’ weights. The only available information are the current weights updating times. The more the numbers of updating for memory cells are, the more reliable the stored values are. As a result, the learning numbers of memory cells are regarded as its credibility. The credibility is higher if the weights’ amendment is smaller. Supposed *f*(*j*) is the learning number of *j*-th memory cell and *m* is the number of activated memory cells in some state, the idea of CA-CMAC is that adjusting errors must be in contrast with the learning numbers of activated memory cells, that is, 1/*m* in Equation (2) can be replaced with ${\left(f(j)+1\right)}^{-1}/\sum_{l=1}^{m}\;{\left(f(j)+1\right)}^{-1}$. Thus learning performance can be improved effectively [14,15]. The specific algorithm can be expressed as:

(3) $$w_{j}\;(t)=w_{j}\;(t-1)+\alpha a_{j}\frac{{\left(f(j)+1\right)}^{-1}}{\sum_{j=1}^{m}\;{\left(f(j)+1\right)}^{-1}}\;\left(\bar{y_{s}}-\sum_{j=1}^{m}\;a_{j}\;w_{j}\;(t-1)\right)$$

## 3. The Proposed Multi-Fault Sensor System Diagnosis Model Based on PCA

### 3.1. Principle of PCA and Signal Forecasting Model

PCA is a statistical analysis technique that can deal with relevance of data. It can reduce the dimensions of original variable *X*, bring effective information in original variable to the full and realize efficient compression of data. Suppose an original variable *X* = [*X*_1_,*X*_2_,…,*X_m_*]*^T^* is an *m*-dimensional random vector and the mean average of each component is zero, that is, *E*(*X*_i_) = 0, (I = 1,2,…,*m*), its covariance matrix can be given by:

(4) $$A=E\left[{\mathrm{XX}}^{T}\right]=\left[\begin{array}{llll} a_{11} & a_{12} & \cdots & a_{1m}\\ a_{21} & a_{22} & \cdots & a_{2m}\\ \vdots & \vdots & \vdots & \vdots \\ a_{m1} & a_{m2} & \cdots \vdots & a_{\mathrm{mm}} \end{array}\right]$$

In Equation (4), *a_ij_* = *E*(*X_i_X_j_*) is the covariance of *X_i_* and *X_j_*. *A* is a positive matrix, and *λ*_1_ ≥ *λ*_2_ ≥…≥ *λ_m_* ≥ 0 are supposed as the eigenvalues of it. *P* is an orthogonal matrix, *P^T^AP* = *Λ*, *Λ = diag*(*λ*_1_, *λ*_2_,*…*,*λ_m_*) (*Λ* is a diagonal matrix). If *Y* = *P^T^X*, then *E*(*YY^T^*) = *E*[*P^T^YY^T^P*] = *P^T^E*[*XX^T^*]*P* = *Λ*, that is, $E\left[Y_{i}Y_{j}\right]=\{\begin{array}{ll} \lambda_{i} & i=j\\ 0 & i\neq j \end{array}$. Therefore, *Y_i_* is irrelevant to *Y_j_*. If *X* is a normal random vector, *Y_i_* and *Y_j_* are also independent. $\lambda_{i}=E\left(Y_{i}^{2}\right)$. The traces of similar matrix are equal, then we can get the equation:

(5) $$\lambda_{1}+\lambda_{2}+\cdots +\lambda_{m}=a_{11}+a_{22}+\cdots +a_{\mathrm{mm}}$$

(6) $$E\left(Y_{1}^{2}\right)+E\left(Y_{2}^{2}\right)+\cdots +E\left(Y_{m}^{2}\right)=E\left(X_{1}^{2}\right)+E\left(X_{2}^{2}\right)+\cdots +E\left(X_{m}^{2}\right)$$

where *λ_1_* *+ λ_2_+…+ λ_m_* is the average energy of *X*. Supposed *a* is a constant between 0 and 1, *β_i_* = *λ*_1_/(*λ*_1_ + *λ*_2_ +…+ *λ_m_*), an integer *s* is choosed to satisfy the equation: *β*_1_ + *β*_2_ +…+ *β_s_* ≥ *a*. The proportion of energy brought by *Y*_1_,*Y*_2_,…,*Y_s_* has exceeded *a*. Therefore *Y*_*s*+1_,*Y*_*s*+2_,…,*Y_m_* can be regarded as random disturbance. Generally, because PCA model can effectively reduce the dimension of input data, *s* is less than *m* and *Y*_1_,*Y*_2_,…,*Y_s_* are the components of *Y*_1_,*Y*_2_,…,*Y_m_* whose significance level is *a*. Each principal component concentrates the common features of all the components in random variable *X*. Obviously, inverse transformation can be expressed as follows:

(7) X=PY

According to the theory of principal component analysis, the orthogonal matrix *P* and principal component matrix *Y* can be obtained by orthogonal transformation of historical data *X* before *k* times, *Y* = *P^T^X*. In addition, according to the eigenvalues of the covariance matrix of *X*, we can find the former *s* principal components *Y*_1_,*Y*_2_,…,*Y_s_* whose significance level is *a*. They can represent the common features of all the components in the random vector *X*, so they should be given priority to be modeled and forecasted.

**Figure 2.**
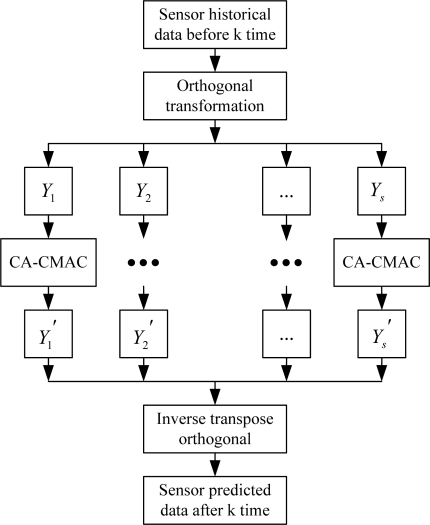
Signal forecasting model based on PCA.

The former *s* principal components *Y*_1_,*Y*_2_,…,*Y_s_* can be forecasted by CA-CMAC neural network to get principal components *Y_i_*’ (I = 1,2,…,*s*). According to the equation *X’ = PY’*, we can get the sensors’ predicted values *X’* that is after *k* cycles. The specific sensor signal forecasting model is shown in Figure 2.

In CA-CMAC neural network training, the inputs of CA-CMAC are the historical values of the principal components *Y_i_* (*i* = 1,2,…,*s*) at the time of (*k*-4, *k*-3, *k*-2, *k*-1), and the desired output is the value of principal components *Y_i_* (*i* = 1,2,…,*s*) at the time *k*. Here the desired output can be also the historical value at time *k*-1, then the inputs of CA-CMAC are historical values at the times (*k*-5, *k*-4, *k*-3, *k*-2), so many training samples can thus be obtained.

Compared with the 5-BP neural network in [8], the 2-RBF network [9,10] and the SOM neural network in [11], the CA-CMAC neural network in Figure 2 has many advantages such as simple structure and high signal forecasting accuracy. Its rapid calculating speed can meet the real-time needs of fault diagnosis systems. In this paper, PCA forecasting model is applied to the detection and isolation of multi-fault sensor system.

### 3.2. Detection and Isolation of a Multi-fault Sensor System Based on PCA

#### (1) Detection of Faulty Sensors

When the sensor system is out of order, a fault can be detected according to some deviation between the actual measured data samples and the predicted values of a statistical model. That is, we can determine whether the system is out of order by the squared prediction error (SPE) between the measured values and normal predicted values of sensors. The estimated error vector is expressed as:

(8) $$e(k)=X(k)-\hat{X}(k)$$

where *e*(*k*) is the error vector of each sensor at *k* time, sensor measured values at *k* time are *X*(*k*) = [*X*_1_(*k*), *X*_2_(*k*),…,*X_n_*(*k)*]. The reconstructed predicted values at *k* time *X̂*(*k*) = [*X̂*_1_(*k*), *X̂*_2_(*k*),⋯, *X̂_n_*(*k*)] can be got by the historical data before *k* time based on PCA, then *SPE* value of sensor system at *k* time can be expressed as follows:

(9) $$\mathrm{SPE}(k)=e^{T}\;(k)e(k)=\sum_{j=1}^{n}\;e_{j}^{2}\;(k)$$

Under normal circumstances, *X̂*(*k*) approximately equals *X*(*k*) and the error *e*(*k*) is small, then the *SPE* value is small too. However, the actual measured values will deviate greatly from the predicted values reconstructed based on PCA when a sensor or more are out of order. Then *E_s_* will increase significantly. The variation curve of *SPE* can be obtained according to Equation (9). If the *SPE* value increases suddenly at some point, it shows that sensor system is out of order at that moment. Regulation of fault detection is defined as:

(10) $$\left\{ \begin{array}{l} \mathrm{SPE}(k)<\delta_{\alpha }-----\mathrm{Free}-\mathrm{fault}\\ \mathrm{SPE}(k)\geq \delta_{\alpha }-----\mathrm{fault} \end{array}\right.$$

In Equation (10), *δ_α_* is the fault threshold of *SPE*.

#### (2) Sensor Fault Isolation Algorithm in Multi-fault Cases

Once the fault of sensor system is monitored, we must find the source of fault accurately in order to exclude the faulty sensors quickly, then the normal values of faulty sensors can be replaced by the predicted values of the model to ensure that the system can operate normally. For the problem of single-fault sensor isolation, a method of linear variable reconstruction for sensor fault isolation has been proposed by Dunia [4,5]. On this basis, we propose an isolation algorithm for multi-fault sensors, fault isolation algorithm of “combined sensors reconstruction”. Specifically, we reconstruct single-sensor signals and multi-sensor signals respectively and replace the sensor signals of actual measured values with the reconstructed values. *SPE* is calculated by Equation (11), and we can determine which sensor is out of order by the hopping of *SPE* curve.

##### ① Reconstruction and Isolation of Sensor Signals

When the sensor system is out of order, the reconstructed value of each sensor at *k* time can be obtained by the measured data before *k* time based on PCA signal reconstructed model, *X̂*(*k*) = [*X̂*_1_(*k*), *X̂*_2_(*k*),⋯, *X̂_j_*(*k*),⋯, *X̂_n_*(*k*)]. Among the sensors’ actual measured values, we just reconstruct the signals of *j*-th sensor at *k* time and define the actual measured values as follows: *X*(*k*) = [*X*_1_(*k*), *X*_2_(*k*),⋯, *X̂_j_*(*k*),⋯, *X_n_*(*k*)](*j* = 1,⋯⋯,*n*), then we can get the following equation from Equations (8) and (9):

(11) $${\bar{\mathrm{SPE}}}_{j}\;(k)=\sum_{i=1}^{n}\;e_{i}^{2}\;(k)$$

Here ${\bar{\mathrm{SPE}}}_{j}\;(k)$ represents the *SPE*(*k*) value after reconstructing the *j*-th variable. If only one sensor is out of order in the system, once the measured values of faulty sensor are reconstructed, ${\bar{\mathrm{SPE}}}_{j}\;(k)$ will be less than the threshold as the fault has been be excluded by reconstruction. If the faulty variable is not reconstructed, ${\bar{\mathrm{SPE}}}_{j}\;(k)$ will still be influenced by the fault and more than the threshold. Accordingly, we can isolate the faulty sensor simply. However, if more than one sensor is out of order, we can not ensure the *SPE* value is less than the threshold by reconstructing only one faulty sensor signal. That is to say, we can not determine the faulty sensors specifically and have to carry out a “combined sensors reconstruction”.

##### ② Reconstruction and Isolation of Multi-Sensor Signals

Suppose that among the *n* sensors to be diagnosed, *m* sensors are out of order. The signals from *m* sensors are reconstructed randomly at each time based on the PCA model. The actual measured values of the *m* sensors at *k* time are replaced with the reconstructed values:

$$X(k)=\left[X_{1}(k),\cdot \cdot \cdot ,{\hat{X}}_{i}(k),\cdot \cdot \cdot ,X_{2}(k),\cdots ,{\hat{X}}_{j}(k),\cdots ,X_{n}(k)\right](i,j=1,\cdot \cdot \cdot \cdot \cdot \cdot ,n)$$

The ${\bar{\mathrm{SPE}}}_{i,j}(k)$ are calculated after reconstructing *m* sensors’ signals according to Equations (8) and (9). If the ${\bar{{\mathrm{SPE}}_{i},}}_{j}(k)\leq \delta_{a}^{2}$, the reconstructed *m* sensors are all out of order. Consequently, the isolation of multi-fault sensors can be realized. However, if ${\bar{{\mathrm{SPE}}_{i},}}_{j}(k)>\delta_{a}^{2}$ is established, the faulty sensors are not isolated completely and combined reconstruction should be done until the result is in accord with ${\bar{{\mathrm{SPE}}_{i},}}_{j}(k)\leq \delta_{a}^{2}$. The maximum numbers of combined reconstruction are $C_{n}^{m}$.

## 4. Simulation Study

### 4.1. Sensors Model

The simulation system is composed of four sensors and the output signals are defined as:

(12) $$\left\{ \begin{array}{l} x_{1}=t+e_{1}\\ x_{2}=t^{2}+t+e_{2}\\ x_{3}=t^{3}+2t+1+e_{3}\\ x_{4}=t^{2}+0.2\;\text{cos}\;2\pi t+e_{4} \end{array}\right.$$

where *e_i_* (*i* = 1,2,3,4) is an independent white noise variable distributed between [−0.1,0.1], *t* is a variable defined between [−1,1] and Δ*t* = 0.005. In the simulation, 400 training data points are adopted. It is assumed that two sensors are out of order. According to the proposed algorithm, *n* = 4, *m* = 2.

### 4.2. Multi-fault Sensor Diagnosis Based on PCA

#### (1) Sensor Fault Detection

According to the signal prediction model based on PCA, the principal component matrix *Y* of historical data matrix *X* = (*x*_1_, *x*_2_, *x*_3_, *x*_4_) can be obtained by orthogonal transformation, so do the first two principal components *Y*_1_,*Y*_2_ whose significance level *α* is 0.9941. Subsequently, the principal components *Y*_1_’, *Y*_2_’ at *k* time are forecasted by the principal components *Y*_1_, *Y*_2_ at the time of (*k*-4, *k*-3, *k*-2, *k*-1). At last, the estimated values *X̂* = (*x̂*_1_, *x̂*_2_, *x̂*_3_, *x̂*_4_) of *X* can be obtained by inverse orthogonal transformation.

**Figure 3.**
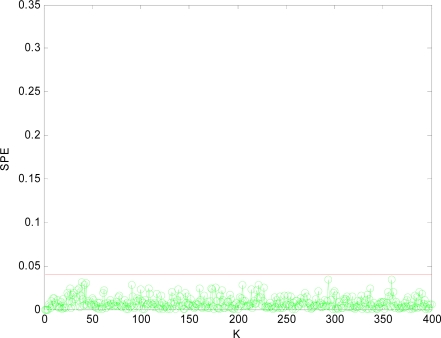
SPE value without fault.

The SPE value under normal circumstance is shown in Figure 3, which shows that the *SPE* value is in a relatively stable state and less than fault threshold (*δ_α_* = 0.04). Thus the sensor data under normal circumstance can be forecasted well by PCA model.

To illustrate the process of fault detection, in this paper, 400 sampling points are used. For variable *x*_1_, a fault is added, which is 14% of its variation range between 150 and 400, and for variable *x*_4_, an added fault is 24% of its variation range between 300 and 400. Then the *SPE* is calculated and the distribution curve is obtained according to Equation (9).

**Figure 4.**
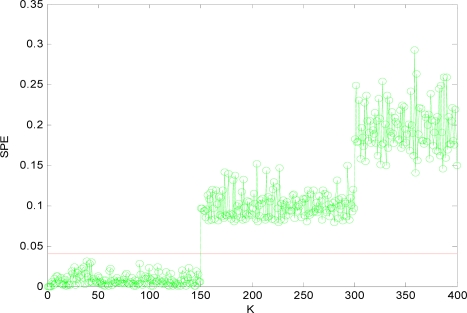
SPE value with fault.

Figure 4 is the *SPE* value of the system when both *x*_1_ and *x*_4_ have a fault. Obviously, starting from the 150th sampling point, the *SPE* value suddenly increases and is more than the fault threshold *δ_α_*. Consequently it can be determined that one sensor is out of order at that time according to the principle of fault detection. At the time of 300th sampling point, the *SPE* value displays another jump, and it shows another sensor is out of order at that time. Accordingly, it can show that not only the sensor system is out of order, but also two sensors (that is *m* = 2) have a fault through the number of jumps of the *SPE* value from Figure 4.

#### (2) Multi-Sensor Fault Isolation

**Figure 5.**
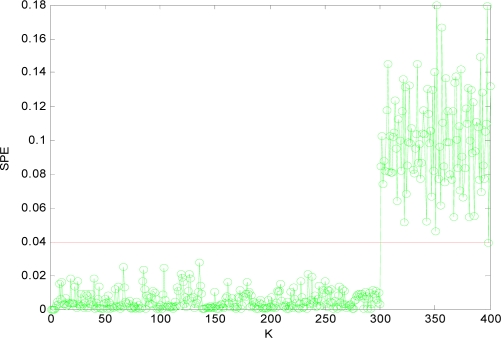
SPE value with sensors 1, 2 reconstructed.

**Figure 6.**
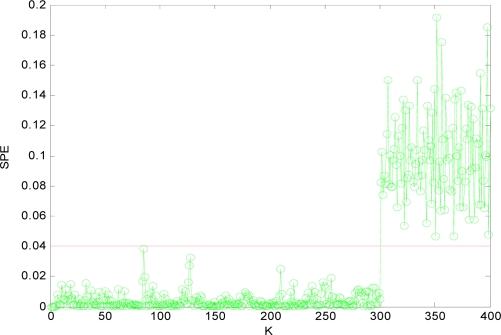
SPE value with sensors 1, 3 reconstructed.

**Figure 7.**
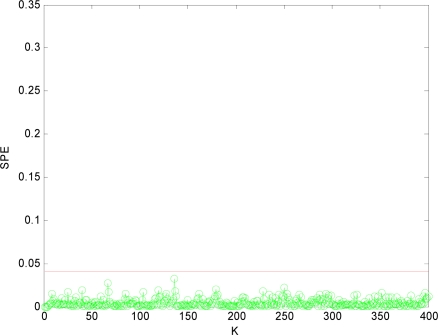
SPE value with the sensors 1, 4 reconstructed.

**Figure 8.**
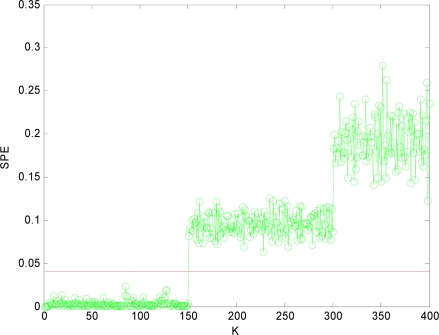
SPE value with sensors 2, 3 reconstructed.

**Figure 9.**
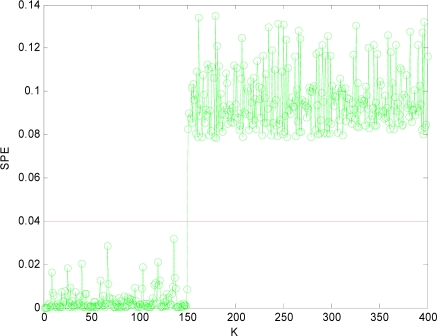
SPE value with sensors 2, 4 reconstructed.

**Figure 10.**
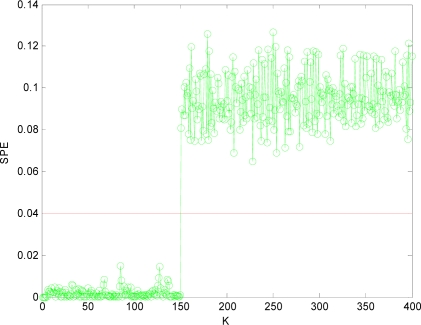
SPE value with sensors 3, 4 reconstructed.

In order to isolate the faulty sensors, in this paper, we adopt the method of reconstructing sensors two by two as *m* = 2. That is, *x*_1_*x*_2_, *x*_1_*x*_3_, *x*_1_*x*_4_, *x*_2_*x*_3_, *x*_2_*x*_4_, *x*_3_*x*_4_ are reconstructed respectively. After the reconstruction, if the *SPE* value of system is less than the fault threshold *δ_α_*, then the two reconstructed sensors at this time are the faulty sensors. The $\bar{{\mathrm{SPE}}_{1,2}}$ value after *x*_1_ and *x*_2_ reconstructed is shown in Figure 5. Then other combinations of variables are reconstructed. Figure 6, Figure 7, Figure 8, Figure 9 and Figure 10 show the values of $\bar{{\mathrm{SPE}}_{1,3}}$, $\bar{{\mathrm{SPE}}_{1,4}}$, $\bar{{\mathrm{SPE}}_{2,3}}$, $\bar{{\mathrm{SPE}}_{2,4}}$, $\bar{{\mathrm{SPE}}_{3,4}}$ after reconstructing *x*_1_*x*_3_, *x*_1_*x*_4_, *x*_2_*x*_3_, *x*_2_*x*_4_, *x*_3_*x*_4_ respectively.

Obviously, after *x*_1_*x*_4_ are reconstructed, the *SPE* value of sensor system is less than the fault threshold. While after reconstructing other combinations, the *SPE* value of sensor system is still more than the fault threshold, which means the fault has not been excluded. Accordingly, it shows that the faulty sensors are *x*_1_ and *x*_4_. That is, sensor 1 and sensor 4 are both out of order and sensor 1 and sensor 4 are the faulty sensors. In Figures 3 to 10, *K* is the time variable (sampling time), the unit of *SPE* is the same as the unit of sensor output variable.

## 5. Comparison among Ca-Cmac, Conventional Cmac and Bp Network

To illustrate the advantage of CA-CMAC neural network adopted in PCA model, take the mentioned PCA model as an example to study the learning effect of the conventional CMAC, CA-CMAC and BP neural networks. CMAC and CA-CMAC use the same network structure. The number of input states is 4, while the number of output states is 1. The BP neural network uses a three-layer network (input layer, hidden layer and output layer), and the number of nodes for each layer is 4, 6 and 1, respectively. The hidden layer node is a sigmoid function, while the output layer node is a linear function. When comparing the convergence rate of PCA with different neural networks, we use mean square error (*MSE*) to describe convergence of neural network:

(13) $$\mathrm{MSE}=\frac{1}{N}\sum_{k=1}^{N}\;{\left(Y_{\mathrm{simuk}}-Y_{t\;\text{arg}\;k}\right)}^{2}$$

where *Y_simuk_* is the output of network, *Y_targk_* is the expected value and *N* is the number of samples.

**Figure 11.**
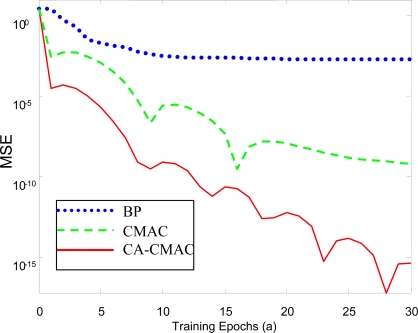
MSE decline curve in the training course of predicting the principal component 1.

The curve in Figure 11 describes the *MSE* decrease with learning cycles in the training process of predicting principal component 1. From curves of *MSE*, it is obvious that the convergence rates of CMAC and CA-CMAC are much faster than that of the BP neural network.

The data in Table 1 is the *MSE* of principal component 1 varying with the cycles in the training process of the three different neural networks. From Table 1, it shows that the convergence rate of CA-CMAC is much faster than that of the BP network, and it is also faster than the CMAC network. Then it can meet the need of the real-time characteristics of a fault diagnosis system.

For each group training data of input and output, all connected weights in the BP neural network should be adjusted, and its calculation demands are increased; in addition, the BP neural network adopts a gradient descent algorithm, so the calculation speed is slow. On thre other hand, the CMAC is a local neural network. It adjusts only part of the weights and adopts the simple *δ* algorithm. Its convergence rate is much faster than BP network and the local minimum does not exist, so it has an obvious advantage in training accuracy and training time. In addition, compared to the conventional CMAC, the conception of credit assignment is introduced to CMAC, so the CA-CMAC can avoid the “corrosion” effect, the correcting errors are distributed in accordance with the cell credibility, then the update of weight is much more rational and effective, the learning time decreases greatly and the real-time characteristic of online learning is improved under the condition of the same approximation accuracy.

## 6. Conclusions

In this paper, a rapid PCA diagnosis model based on CA-CMAC for multi-fault diagnosis is proposed. Simulation results show that the faulty sensors can be isolated by the method of “combined reconstruction”. CA-CMAC provides a noticeable improvement in learning speed and accuracy in comparison to the conventional CMAC and BP neural networks. The proposed algorithm is practical and effective.

## Figures and Tables

**Table 1.** *MSE* values by using different neural networks to train principal component 1.

Neural Network	Training Cycle					
	0	1	2	3	4	5

BP	2.77223	2.67898	0.52841	0.21499	0.03188	0.02098
CMAC	2.90312	0.29E-02	0.54E-02	0.51E-02	0.30E-02	0.12E-02
CA-CMAC	2.90312	0.31E-04	0.51E-04	0.31E-04	0.10E-04	0.21E-05

Neural Network	Training Cycle					
	6	7	8	9	10	11

BP	0.01487	0.01156	0.00580	0.00391	0.00316	0.00282
CMAC	0.34E-03	0.66E-04	0.55E-05	0.23E-06	0.25E-05	0.29E-05
CA-CMAC	0.29E-06	0.24E-07	0.79E-09	0.30E-09	0.74E-09	0.64E-09
